# Decreased consumption of natural rewards in rhesus monkeys with prolonged methamphetamine abstinence

**DOI:** 10.1192/j.eurpsy.2026.10479

**Published:** 2026-07-31

**Authors:** J. Zhou

**Affiliations:** University of Tübingen, Tübingen, Germany

## Abstract

**Introduction:**

Relapse to drug use is a major clinical challenge in the treatment of addictive disorders, including psychostimulant use and may be exacerbated by reduced sensitivity to natural, non-drug reward. Given the relatively limited set of outcomes, and short withdrawal time in rodent studies, we conducted a more detailed assessment of the response to natural rewards in methamphetamine (METH) naive versus exposed monkeys during long-term abstinence.

**Objectives:**

This study introduced an improved sucrose preference test (iSPT) to assess natural reward seeking and consumption in monkeys with long-term abstinence after methamphetamine (METH) use. The test was administered to sixteen naive monkeys and five METH exposed monkeys that had been abstinent for at least 3 months.

**Methods:**

Sixteen naive monkeys without being exposed to METH and five METH exposed monkeys abstinent for at least three months were studied during a withdrawal period and subjected to improved sucrose preference test (iSPT) and sucrose preference test (SPT). The improved paradigm assessed sucrose preference scores and three behavioral indicators extracted from video recordings. These behavioral indicators were supposed to reflect different dimensions of approach behaviors and consumption in both animal groups.

**Results:**

METH exposed monkeys showed a lower sucrose preference score in both the iSPT (z = -2.10, p = 0.036) and the sucrose preference test (z = -2.61, p =0.009). The sucrose preference score was significantly correlated with the latency of the establishment of stable sucrose-preference (r = -0.76, df = 46, p < 0.001) but not with the other variables. Furthermore, water-sucrose switch latency and switch times were significantly negatively correlated (r = -0.50, df = 20, p = 0.02).

**Image 1:**
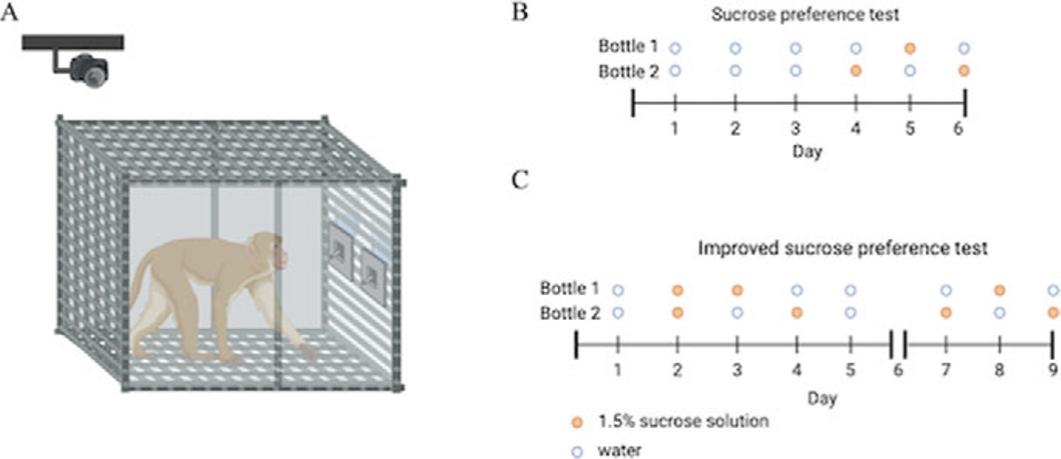
Image 1: Long description.

**Image 2:**
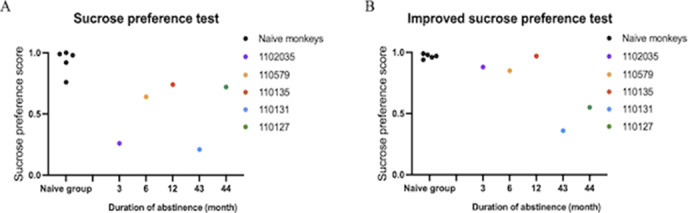
Image 2: Long description.

**Image 3:**
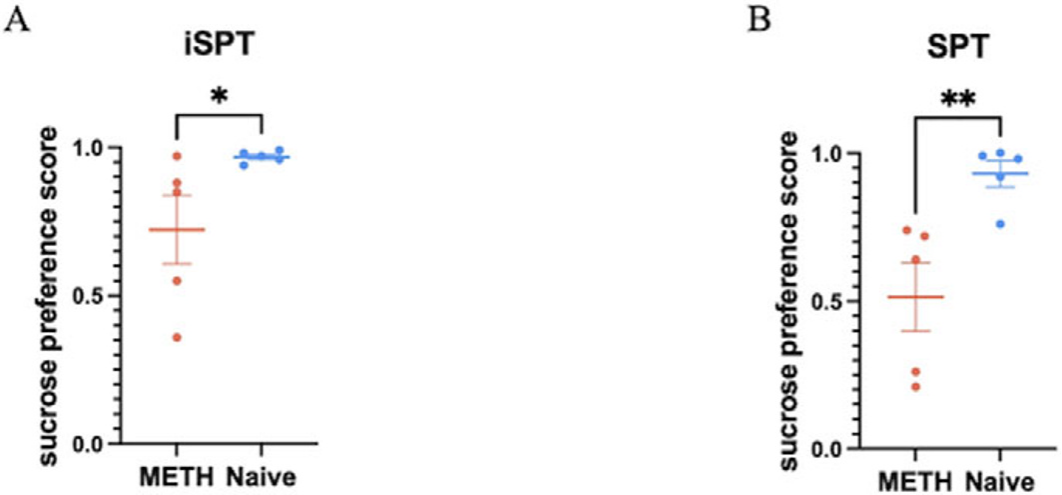
Image 3: Long description.

**Conclusions:**

These results show reductions in natural reward consumption during long-term methamphetamine abstinence.

**Disclosure of Interest:**

None Declared

## Long descriptions

### Image 1: Long description

Panel A shows a monkey inside a wire mesh cage. Above the cage is a camera mounted on a rail. On the right wall of the cage, there are two square ports with drinking spouts.

Panel B is titled Sucrose preference test. It features a horizontal timeline for Day 1 through Day 6. Above the timeline are two rows of circles representing Bottle 1 and Bottle 2. Blue open circles represent water and orange solid circles represent 1.5% sucrose solution. On days 1, 2, 3, and 6, both bottles contain water. On day 4, Bottle 2 contains sucrose. On day 5, Bottle 1 contains sucrose.

Panel C is titled Improved sucrose preference test. It features a horizontal timeline for Day 1 through Day 9 with a break between Day 5 and Day 7. On Day 1, both bottles contain water. On Day 2, both bottles contain sucrose. On Day 3, Bottle 1 contains sucrose and Bottle 2 contains water. On Day 4, Bottle 1 contains water and Bottle 2 contains sucrose. On Day 5, both bottles contain water. After the break, on Day 7, Bottle 1 contains water and Bottle 2 contains sucrose. On Day 8, Bottle 1 contains sucrose and Bottle 2 contains water. On Day 9, both bottles contain sucrose.

Navigate back to Image 1:.

### Image 2: Long description

Panel A is titled Sucrose preference test. The y-axis is Sucrose preference score ranging from 0.0 to 1.0. The x-axis is Duration of abstinence month with categories Naive group, 3, 6, 12, 43, and 44. In the Naive group, six black dots are clustered between 0.75 and 1.0. For the individual monkeys, the scores are: monkey 1 1 0 2 0 3 5 at 3 months is approximately 0.25; monkey 1 1 0 5 7 9 at 6 months is approximately 0.65; monkey 1 1 0 1 3 5 at 12 months is approximately 0.75; monkey 1 1 0 1 3 1 at 43 months is approximately 0.2; and monkey 1 1 0 1 2 7 at 44 months is approximately 0.7.

Panel B is titled Improved sucrose preference test. It uses the same axes and legend as Panel A. In the Naive group, six black dots are tightly clustered near 1.0. For the individual monkeys, the scores are: monkey 1 1 0 2 0 3 5 at 3 months is approximately 0.85; monkey 1 1 0 5 7 9 at 6 months is approximately 0.8; monkey 1 1 0 1 3 5 at 12 months is approximately 0.95; monkey 1 1 0 1 3 1 at 43 months is approximately 0.35; and monkey 1 1 0 1 2 7 at 44 months is approximately 0.55.

Navigate back to Image 2:.

### Image 3: Long description

Panel A is titled i S P T. The y-axis is sucrose preference score ranging from 0.0 to 1.0. The x-axis compares two groups. The METH group shows a wide distribution of red data points with a mean around 0.7 and large error bars. The Naive group shows tightly clustered blue data points near 1.0. A bracket with a single asterisk indicates a significant difference between the groups.

Panel B is titled S P T. The y-axis is sucrose preference score ranging from 0.0 to 1.0. The x-axis compares two groups. The METH group shows red data points with a mean around 0.5 and moderate error bars. The Naive group shows blue data points clustered near 0.9. A bracket with two asterisks indicates a highly significant difference between the groups.

Navigate back to Image 3:.

